# Polyethylenimine-Conjugated Hydroxyethyl Cellulose for Doxorubicin/Bcl-2 siRNA Co-Delivery Systems

**DOI:** 10.3390/pharmaceutics15020708

**Published:** 2023-02-20

**Authors:** Jiwon Park, Seoyoung Kim, Tae-il Kim

**Affiliations:** 1 Department of Agriculture, Forestry and Bioresources, College of Agriculture and Life Sciences, Seoul National University, 1 Gwanak-ro, Gwanak-gu, Seoul 08826, Republic of Korea; 2 Department of Biosystems & Biomaterials Science and Engineering, College of Agriculture and Life Sciences, Seoul National University, 1 Gwanak-ro, Gwanak-gu, Seoul 08826, Republic of Korea; 3 Research Institute of Agriculture and Life Sciences, Seoul National University, 1 Gwanak-ro, Gwanak-gu, Seoul 08826, Republic of Korea

**Keywords:** gene/drug delivery systems, hydroxyethyl cellulose, polyethylenimine, serum stability, doxorubicin, Bcl-2 siRNA, drug resistance, anti-cancer effect

## Abstract

Hydroxyethyl cellulose (HEC), widely known for its biocompatibility and water solubility, is a polysaccharide with potential for pharmaceutical applications. Here, we synthesized polyethylenimine2k (PEI2k)-conjugated hydroxyethyl cellulose (HECP2k) for doxorubicin/Bcl-2 siRNA co-delivery systems. HECP2ks were synthesized by reductive amination of PEI2k with periodate-oxidized HEC. The synthesis of the polymers was characterized using ^1^H NMR, ^13^C NMR, primary amine quantification, FT-IR, and GPC. Via agarose gel electrophoresis and Zeta-sizer measurement, it was found that HECP2ks condensed pDNA to positively charged and nano-sized complexes (100–300 nm, ~30 mV). The cytotoxicity of HECP2ks was low and HECP2k 10X exhibited higher transfection efficiency than PEI25k even in serum condition, showing its high serum stability from ethylene oxide side chains. Flow cytometry analysis and confocal laser microscopy observation verified the superior cellular uptake and efficient endosome escape of HECP2k 10X. HECP2k 10X also could load Dox and Bcl-2 siRNA, forming nano-particles (HECP2k 10X@Dox/siRNA). By median effect analysis and annexin V staining analysis, it was found that HECP2k 10X@Dox/siRNA complexes could cause synergistically enhanced anti-cancer effects to cancer cells via induction of apoptosis. Consequently, it was concluded that HECP2k possesses great potential as a promising Dox/Bcl-2 siRNA co-delivery carrier.

## 1. Introduction

A gene delivery system is a method of introducing new genes or suppressing over-expressed genes by transferring external genetic materials. An ideal gene delivery system requires high transfection efficiency, low cytotoxicity, and cell specificity [1]. Gene delivery carriers can be classified into viral and non-viral vectors. While viral vectors can bind with cell membranes efficiently and show high transfection efficiency, they possess several problems such as evoking immune responses and showing toxicity to host cells. In addition, high cost and difficulties in mass production limit their pharmaceutical applications. In contrast, despite relatively low transfection efficiency, non-viral vectors are known to be safe, compared to viral vectors. Additionally, it is easy to introduce the desired functionality to non-viral vectors, especially polymeric gene delivery carriers for the improvement of gene delivery efficiency [2,3]. Therefore, a lot of cationic polymers have been developed for gene delivery systems. The cationic polymers can form complexes with anionic genes via electrostatic interaction and easily adsorb to negatively charged cell membranes [4].

Polysaccharides are one of the candidates for efficient gene/drug delivery systems due to their high biocompatibility, inherent bio-functionality, biodegradability, chemically modifiable functional groups, and abundance [5]. Generally, cationic polymers such as PEI or other functionalities have been introduced to polysaccharides for gene delivery systems by chemical modifications of functional groups [6].

Hydroxyethyl cellulose (HEC) is a cellulose derivative where hydroxyl groups on C2, C3, or C6 are substituted to hydroxyethyl groups. The synthesis of HEC is conducted via dissolution of cellulose in sodium hydroxide and reaction with ethylene oxide [7]. HEC has additional advantages for biomedical applications. First, HEC is replenishable because its raw material is cellulose, the most abundant polysaccharide in nature. Second, HEC has high water solubility; thus, it can be well perfused and does not require organic solvents in the synthesis process [8,9]. Additionally, the side chain unit of HEC has the same structure as a repeating unit of poly(ethylene glycol) (PEG), commonly used for enhancing serum stability in biomedical fields [10,11]. Therefore, it is reasonable to suppose that HEC can possess high serum stability as the main material for gene/drug delivery systems.

Despite these advantages, there are few cases of developing HEC as an efficient drug/gene delivery carrier. Himmelein et al. developed HEC-based hydrogel containing cyclodextrin vesicles as 3D junctions [12]. Therapeutic agents could be loaded inside the vesicles and controlled release could be induced with the biocompatibility of the components. Ho et al. investigated HEC-based gel containing metronidazole-loaded lipid nanoparticles for buccal mucosal drug delivery [13]. Their hydrogel exhibited a sustained release pattern and enhanced antimicrobial treatment activity. Fayazpour et al. researched polyquaternium-4 (PQ-4) and polyquarternium-10 (PQ-10), cationic hydroxyethyl cellulose materials, to use as gene delivery carriers [14]. They aimed to characterize the properties of PQ-4/pDNA and PQ-10/pDNA nanoparticles and noticed that the sugar monomers of PQ-4 and PQ-10 were substituted with PEG. However, PQ-4/pDNA and PQ-10/pDNA nanoparticles exhibited poor transfection efficiency because of their electrically neutral surface and too strong binding of pDNA, respectively. Consequently, it can be worthwhile to develop novel gene/drug delivery systems with excellent transfection efficiency while taking advantage of HEC.

In this study, PEI2k-conjugated HEC (HECP2k) was developed for anti-cancer drug, doxorubicin (Dox), and Bcl-2 siRNA co-delivery systems. Notably, to our knowledge, there is no reported research to develop HEC as a gene/drug co-delivery system. This may be because appropriate chemistry for introducing the cationic moiety has not yet been developed. Here, we used simple chemistries such as periodate oxidation and reductive amination to introduce the cationic moiety to HEC in mild conditions (aqueous solution, room temperature). Via this strategy, we expected to achieve excellent drug/gene delivery efficiency, controlling the conjugation ratio of the cationic moieties [15]. After the synthesis of HECP2ks, the physicochemical properties of the polymers and complexes were analyzed. Finally, cytotoxicity, transfection efficiency, serum stability, intracellular trafficking, and synergistic anti-cancer effect by inducing the apoptosis of HECP2k were investigated for drug/gene co-delivery systems.

## 2. Materials and Methods

### 2.1. Materials

Hydroxyethyl cellulose (HEC, viscosity: ~145 mPa∙s in 1% H_2_O, average molecular weight: ~250,000 Da, provided by the manufacturer), branched polyethylenimine (PEI25k, 25 kDa), agarose, gel loading solution, ethidium bromide, dimethyl sulfoxide (DMSO), MicroBCA™ protein assay kit, 4′,6-diamidino-2-phenylindole (DAPI), and triethylamine (TEA) were purchased from Sigma-Aldrich (St. Louis, MO, USA). Branched polyethylenimine (PEI, 2 kDa) was purchased from Polysciences (Warrington, PA, USA). Sodium metaperiodate (NaIO_4_) was purchased from Alfa-aesar (Haverhill, MA, USA). Sodium tetrahydroborate (NaBH_4_) and HPLC water were purchased from Duksan (Ansan, Korea). Formic acid was purchased from Merck (Darmstadt, Germany). Triazolyl blue tetrazolium bromide (MTT) was purchased from Gold Biotechnology (St. Louis, MO, USA). pDNA (pCN-Luci) was amplified by using Dyne DH5α Chemically Competent *E.coli* ver.2 (DYNEBIO, Sungnam, Korea) and purified by using NucleoBond Xtra Maxi (Macherey-Nagle, Duren, Germany). Dulbecco’s Modified Eagle Medium (DMEM) and Dulbecco’s phosphate buffered saline (DPBS) were purchased from Cytiva (Marlborough, MA, USA). DMEM + GlutaMAX (GMX), fetal bovine serum (FBS), penicillin–streptomycin (P–S), and trypsin-EDTA were purchased from Gibco (Waltham, MA, USA). Luciferase assay system and reporter lysis buffer were purchased from Promega (Madison, WI, USA). YOYO-1 iodide and Lysotracker Red DND-99 were purchased from Invitrogen (Waltham, MA, USA). Doxorubicin hydrochloride (Dox∙HCl) was purchased from Cayman Chemical (Ann Arbor, MI, USA). Alexa Fluor 488 Annexin V/Dead cell apoptosis kit was purchased from Life Technologies (Carlsbad, CA, USA). Bcl-2 siRNA (5′-GUGAAGUCAACAUGCCUGC-3′ (sense), 5′-GCAGGCAUGUUGACUUCAC-3′ (anti-sense)) was purchased from Bioneer (Daejeon, Korea).

### 2.2. Synthesis of Polyethylenimine (PEI2k)-Conjugated Hydroxyethyl Cellulose (HECP2k)

Prior to HECP2k synthesis, HEC was oxidized by periodate ions. Sodium metaperiodate solution (water, one molar equivalent of the sugar units of HEC) was added to the HEC solution (water) with continuous stirring. After 2 h of reaction under 25 °C in the dark, the solution was dialyzed against ultra-pure water for 48 h using a dialysis membrane (MWCO: 3.5 kDa). The product, oxidized-HEC (OxHEC) was obtained after lyophilization.

Then, HECP2k was synthesized by reductive amination of OxHEC and PEI2k. Various amounts of PEI2k solution (water, HEC:PEI2k = 1:1, 1:5, or 1:10 *w*/*w*, respectively) were prepared and OxHEC solutions (water) were added to the PEI solution with continuous stirring. After 24 h of reaction under 25 °C in the dark, excessive sodium tetrahydroborate was added to the reaction mixture with continuous stirring. After an additional 24 h of reaction 25 °C in the dark, the solutions were dialyzed against ultra-pure water for 72 h. The products, HECP2k 1X, 5X, or 10X were obtained after lyophilization. The synthesis scheme of HECP2k is shown in Figure S1.

### 2.3. Characterization of HECP2k

The synthesis of HECP2k was confirmed by ^1^H NMR and ^13^C NMR, respectively (600 MHz, AVANCE 600, Brucker, Germany). D_2_O was used as the solvent. Each sample was prepared at a concentration of 10 mg/mL for ^1^H NMR and 50 mg/mL for ^13^C NMR.

The synthesis of the polymers was also confirmed using an ATR (attenuated total reflectance) FT-IR spectrometer (Nicolet 6700, Thermo Scientific, Waltham, MA, USA). The measurements were performed in the wavenumber range of 4000–650 cm^−1^ with 8 cm^−1^ by 32 times.

The primary amine (1° amine) quantification of HECP2k was performed via fluorescamine assay. The calibration curve was prepared by using PEI2k as a standard. The primary amine contents of the PEI2k (molar amounts of 1°:2°:3° amine = 40:36:24) were provided by the manufacturer. We reacted 75 μL of HECP2k sample solution (10 μg/mL, PBS) and 25 μL of fluorescamine solution (3 mg/mL, DMSO) for 15 min at 25 °C. The fluorescence was measured at Ex/Em = 380/470 nm with a microplate reader (Synergy H1, BioTek, Winooski, VT, USA). After the measurements, the molar amount of primary amine per 1 g of HECP2k was calculated for each sample and compared with ^1^H NMR results.

The molecular weights of the polymers were measured via gel permeation chromatography (GPC, YL-9100 HPLC System, Youngin Chromass, Anyang, Korea). Each sample was prepared at a concentration of 10 mg/mL. We used 1% formic acid as an eluent. Poly(ethylene glycol)s with various molecular weights were used as standards for analysis. The assay was run on an Ultrahydrogel 250 column (Waters, Milford, MA, USA) at 0.6 mL/min of flow rate.

### 2.4. Characterization of HECP2k/pDNA Polyplexes

The pDNA condensing ability of HECP2k was examined via agarose gel electrophoresis. HECP2k/pDNA polyplexes were prepared in Hepes buffer (pH 7.4) at various weight ratios ranging from 0.1 to 0.9 for HECP2k 1X and from 0.1 to 0.5 for HECP2k 5X and 10X, respectively. Agarose gel (0.7% *w*/*v*) containing ethidium bromide solution (0.5 μg/mL) was prepared in TAE (Tris-Acetate-EDTA) buffer. After 30 min of incubation at room temperature, the samples were electrophoresed at 80 V for 12 min using Mupid-2plus (Takara Bio, Kusatsu, Japan). The pDNA bands were visualized with a GelDoc XRS+ gel documentation system (Bio-Rad, Hercules, CA, USA).

Average sizes and zeta-potential values of the polyplexes were measured by using a Zeta-sizer Nano ZS (Malvern Instruments, Malvern, UK). The polyplex solutions (4 μg pDNA, 1 mL) were prepared in ultra-pure water at various weight ratios ranging from 0.1 to 30. After 30 min of incubation at 25 °C, the measurements were performed 3 times.

### 2.5. Cell Culture

Human cervical adenocarcinoma cells (HeLa, origin: human cervix, uterine, Korean Cell Line Bank) and human hepatocellular carcinoma cells (HepG2, origin: human liver, Korean Cell Line Bank) were maintained using cell media (DMEM + GlutaMAX and DMEM, respectively) supplemented with 10% of FBS and 1% of penicillin–streptomycin (P–S) in a humidified atmosphere containing 5% of CO_2_ at 37 °C.

### 2.6. Cell Metabolic Activity Analysis with MTT Assay

The cytotoxicity of HECP2k was estimated via MTT assay. The cells (HeLa and HepG2) were seeded on a 96-well cell culture plate at a density of 1 × 10^4^ cells/well in 100 μL of culture medium. As the cells achieved 70–80% confluency after 24 h of incubation, the cells were treated with HECP2k solutions (serum-free medium) with various concentrations from 10 μg/mL to 100 μg/mL for 4 h. PEI25k was used as a control. Then, the medium was exchanged with fresh medium containing 10% FBS. After 24 h of incubation, 25 μL of MTT solution (2 mg/mL, DPBS) was added to each well and further incubated for 2 h. The medium was removed and 150 μL of DMSO was added to each well to dissolve formazan crystals formed by cell metabolism. The absorbance was measured at 570 nm by using a microplate reader. The results were presented as relative cell viability (RCV, %) by calculating the percentage values relative to the untreated cell control group.

### 2.7. Transfection Efficiency with Luciferase Transgene Expression Assay

The transfection efficiency of HECP2k was estimated with luciferase transgene expression assay. The HeLa cells were seeded on a 24-well cell culture plate at a density of 5 × 10^4^ cells/well in 500 μL of culture medium and incubated for 24 h until the cells achieved 70–80% confluency. Prior to treatment, the medium was exchanged with 450 μL of fresh medium (both serum-free medium and serum-containing medium). Then, the cells were treated with HECP2k/pDNA polyplex solutions (50 μL, 0.5 μg of pDNA/well) with various weight ratios. PEI25k/pDNA polyplex (weight ratio = 1) was used as a positive control. After 4 h of incubation, the medium was exchanged with fresh medium containing 10% FBS. Each medium was aspirated off after 48 h of incubation. The cells were rinsed with DPBS and lysed with 120 μL of reporter lysis buffer. The cell lysates were centrifuged (14,000 RPM, 10 min, 4 °C). We added 20 μL of the supernatant to a white 96-well plate and 100 μL of the luciferase assay buffer was dispensed to each well. The luminescence was measured by using a microplate reader. To quantify the amounts of protein in the supernatant, MicroBCA protein assay was performed. We dispensed 130 μL of ultra-pure water, 150 μL of BCA reagent, and 20 μL of the supernatant to a 96-well plate. After 2 h of incubation at 37 °C, the absorbance was measured at 562 nm by using a microplate reader. The results were presented in terms of relative luminescence unit per unit mg of cellular protein (RLU/mg protein).

### 2.8. Cellular Uptake Measurement with Flow Cytometry

To estimate cellular uptake of the HECP2k/pDNA polyplex, flow cytometry was used. HeLa cells were seeded on a 6-well cell culture plate at a density of 2 × 10^5^ cells/well in 2 mL of medium and incubated for 24 h until the cells achieved 70–80% confluency. After exchange with fresh serum-free medium, the cells were treated with polyplex solutions (1 μg of pDNA/well, pDNA labeled with YOYO-1 iodide (1 dye molecule per 50 base pairs of nucleotides)). After 4 h, the medium was aspirated off and the cells were rinsed with ice-cold DPBS. Trypan blue solution (1 mg/mL) was treated to quench the unnecessary fluorescence of dye molecules at the cell membrane surface. The cells were rinsed with DPBS twice. After 2 min of trypsinization, the detached cells were re-suspended in DPBS. The cellular uptake of fluorescence-labeled polyplexes and the mean fluorescence were examined by using a BD Accuri C6 flow cytometer (Becton Dickinson, San Jose, CA, USA) at a minimum of 1 × 10^4^ cells gated per sample. The analysis was performed by using BD Accuri C6 software.

### 2.9. Intracellular Trafficking Visualization

Intracellular trafficking of the polyplexes was observed by using a confocal laser scanning microscope (CLSM). HeLa cells were seeded on a confocal dish at a density of 3 × 10^5^ cells/dish in 3 mL of medium and incubated for 24 h until the cells achieved 70–80% confluency. After exchange with fresh serum-free medium, the cells were treated with HECP2k/pDNA polyplex solutions (1.5 μg of pDNA/dish, pDNA labeled with YOYO-1 iodide) with optimal weight ratio (50). PEI25k/pDNA polyplex (weight ratio = 1) was used as a control. After 4 h of incubation, the medium was exchanged with fresh medium containing 10% FBS and further incubated for 2 h or 4 h. Then, acidic organelles were stained using Lysotracker Red DND-99 solution (100 nM) and the cells were fixed with 4% paraformaldehyde. Nuclei were labeled with DAPI solution (0.125 μg/mL). After sufficient rinsing of cells, visualization proceeded with a confocal laser scanning microscope (SP8 X STED, Leica, Wetzlar, Germany) and the images were processed with LAS X software.

### 2.10. Serum Stability

To estimate the serum stability of the HECP2k polyplex, transfection experiments were performed in various serum concentration conditions. HeLa cells were seeded on a 24-well cell culture plate at a density of 5 × 10^4^ cells/well and incubated for 24 h until they achieved 70–80% confluency. Before transfection, the medium of each well was exchanged with fresh medium containing various FBS concentrations (10, 30, or 50%, respectively). Then, polyplex solutions (0.5 μg of pDNA/well) were added to each well with optimal ratios (weight ratio = 50). PEI25k was used as a control. After 4 h of treatment, the medium was exchanged with fresh medium containing 10% FBS, and the cells were further incubated for 48 h. Then, a luciferase transgene expression assay was performed with a method identical to above. The results were formatted in terms of relative luminescence unit per unit mg of cellular protein (RLU/mg protein).

### 2.11. Evaluation of Doxorubicin Loading and siRNA Complexation

Dox∙HCl was dissolved in DMSO at a final concentration of 5 mg/mL. We added a 2 molar excess of Triethylamine (TEA) to the Dox∙HCl solution with continuous stirring for 24 h. After desalting, Dox solution was added dropwise to the HECP2k solution (5 mg/mL, DMSO:water = 1:9) with continuous stirring for 90 min. Then, the mixture solution was added dropwise to 10 mL of ultra-pure water with continuous stirring for 24 h and dialyzed against ultra-pure water for 6 h using a dialysis membrane (MWCO: 2 kDa) for 6 h. The product, HECP2k@Dox, was obtained after lyophilization.

The absorbance of HECP2k@Dox solution (DMSO:water = 1:9) was measured at 480 nm by using a microplate reader. The loaded Dox amount was determined according to the prepared calibration curve of Dox solution. Drug loading content (DLC) and drug loading efficiency (DLE) were calculated as the following formula.

(1) $$\mathrm{DLC}\;\left(\%\right)=\frac{\mathrm{Weight}\;\mathrm{of}\;\mathrm{loaded}\;\mathrm{Dox}}{\mathrm{Weight}\;\mathrm{of}\;\mathrm{HEPC}2k}\times 100$$

(2) $$\mathrm{DLE}\;\left(\%\right)=\frac{\mathrm{Weight}\;\mathrm{of}\;\mathrm{Loaded}\;\mathrm{Dox}}{\mathrm{Weight}\;\mathrm{of}\;\mathrm{total}\;\mathrm{Dox}\;\mathrm{for}\;\mathrm{loading}}\times 100$$

Bcl-2 siRNA complexation was conducted to prepare HECP2k/siRNA polyplexes and HECP2k@Dox/siRNA complexes. HECP2k and HECP2k@Dox solutions were mixed with Bcl-2 siRNA solution at a weight ratio of 50. The complexes were incubated at 25 °C for 30 min. Average sizes and zeta-potential values of the complexes were measured by using a Zeta-sizer Nano ZS.

### 2.12. Anti-Cancer Effect Analysis with MTT Assay

The anti-cancer effect of the HECP2k@Dox/siRNA was estimated via MTT assay. The cells were seeded on a 96-well cell culture plate at a density of 1 × 10^4^ cells/well in 100 μL of medium. As the cells achieved 70–80% confluency after 24 h of incubation, the cells were treated for 4 h in serum-free medium with Dox (Dox∙Hcl), siRNA, PEI25k/siRNA, HECP2k/siRNA, HECP2k@Dox, or HECP2k@Dox/siRNA, respectively. DLC value and the optimal weight ratio of polymer/pDNA (50 for HECP2k and 1 for PEI25k) were considered to adjust the concentrations of Dox and siRNA: low (0.5 μg/mL siRNA and 3.7 μg/mL Dox), mid (1 μg/mL siRNA and 7.4 μg/mL Dox), and high (2 μg/mL siRNA and 14.8 μg/mL Dox). Then, the medium was exchanged with fresh medium containing 10 % FBS. After 24 h of incubation, MTT assay was performed in a method identical to the above. The results were formatted as relative cell viability (RCV, %) by calculating the percentage values relative to the untreated cell control group. Statistical analysis was performed through one-way ANOVA and a Bonferroni post hoc test. Statistical significance is presented as follows (*p* < 0.05 *, *p* < 0.01 **, and *p* < 0.001 ***).

### 2.13. Apoptosis Analysis with Annexin V Staining

Apoptosis induction was investigated via Annexin V staining using a Alexa Fluor 488 Annexin V/Dead cell apoptosis kit. HepG2 cells were seeded on a 6-well cell culture plate at a density of 2 × 10^5^ cells/well in 2 mL of medium containing 10% FBS and 1% P–S and incubated for 24 h until the cells achieved 70–80% confluency. After exchange with fresh serum-free medium, the cells were treated with HECP2k@Dox/siRNA nano-complex solution (2 μg/mL siRNA and 14.8 μg/mL Dox) for 4 h. Then, the medium was exchanged with fresh medium containing 10% FBS. After 24 h of incubation, the cells were rinsed with iced DPBS twice. After 2 min of trypsinization, the detached cells were re-suspended in 500 μL of DPBS. To label the cells, 5 μL of Alexa Fluor 488 solution and 10 μL of propidium iodide (PI) solution were added to the cell suspension. After 15 min of incubation in dark condition, the cells were analyzed using a BD Accuri C6 flow cytometer with BD Accuri^TM^ C6 software (Version:1.0.264.21, Becton Dickinson, San Jose, CA, USA).

## 3. Results and Discussion

### 3.1. Synthesis of HECP2k

It is well known that PEI can show high transfection efficiency with endosome escape ability from the proton sponge effect, condensing nucleic acids and forming positively charged nanoparticles [16,17]. However, high molecular weight PEI also shows severe cytotoxicity. Therefore, various strategies for reducing its cytotoxicity have been reported, such as conjugation with natural polymers or using low molecular weight PEI [18,19]. In this work, we tried to introduce low molecular weight PEI2k to the polysaccharide, HEC, as a cationic moiety. In order to synthesize HECP2k, HEC was oxidized via periodate oxidation with sodium metaperiodate in aqueous solution instead of using coupling agents or crosslinkers. Aldehyde groups, which can react with amines, would be introduced by cleaving the vicinal diols in anhydroglucose units of HEC [20,21]. After obtaining oxidized HEC (OxHEC), PEI2k was conjugated with various weight ratios (OxHEC:PEI2k) of 1:1, 1:5, and 1:10, also in aqueous condition, by the formation of Schiff base (-C=N-), which was subsequently reduced to a stable -C-NH- bond by sodium tetrahydroborate. Unreacted PEI2k could be almost 100% removed by dialysis with a dialysis membrane (MWCO 3.5 kDa). Finally, HECP2ks with three different feed ratios of 1:1, 1:5, and 1:10 were obtained and named HECP2k 1X, 5X, and 10X, respectively.

### 3.2. Structural Analysis with ^1^H NMR and Primary Amine Quantification

**Figure 1:**
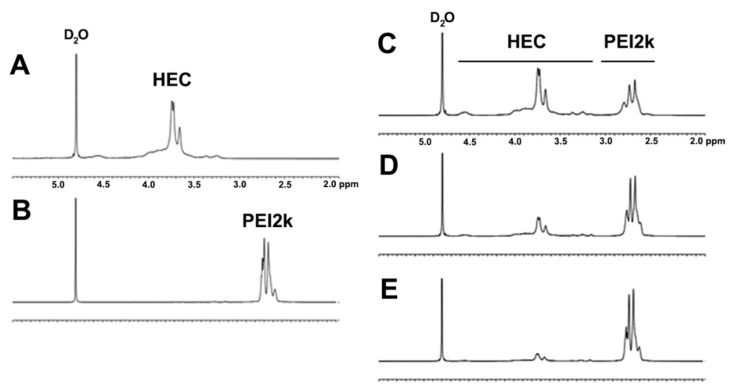
^1^H NMR spectra of (**A**) HEC, (**B**) PEI2k, (**C**) HECP2k 1X, (**D**) HECP2k 5X, and (**E**) HECP2k 10X in D_2_O solvent.

The chemical structures of the HECP2ks were analyzed via ^1^H NMR (Figure 1). Proton peaks of HEC and PEI2k were observed at δ 3.1–4.7 and δ 2.4–3.0, respectively. As the feed ratio increased, the relative ratio of proton peaks of PEI2k to HEC increased, as expected. The NMR spectra of HECP2k 1X, 5X, and 10X confirmed that conjugation between HEC and PEI was successfully performed.

Assuming that the total molar substitution of ethylene oxide side chains is 2.5 and comparing all the proton peaks of HEC and PEI2k, it was calculated that one molecule of PEI2k was grafted to every 18.4, 5.8, and 2.4 sugar molecules of HECP2k 1X, 5X, and 10X, respectively. Converting this value into the number of primary amine moles per unit mass of the polymer, it was estimated that 2.7 mmol/g, 5.2 mmol/g, and 7.0 mmol/g of primary amines were present in HECP2k 1X, 5X, and 10X, respectively. Therefore, HECP2ks with various compositions were synthesized in a controllable manner as expected.

Primary amine quantification by using fluorescamine was further performed to validate the chemical structures analysis result of HECP2ks via ^1^H NMR. As a result, the molar amount of primary amine per unit mass of the polymer was measured as 1.48 mmol/g, 2.13 mmol/g, and 3.86 mmol/g for HECP2k 1X, 5X, and 10X, respectively. The tendency of increasing the primary amine amount was correlated with the ^1^H NMR result. The discordance between these results may be mainly derived from the difficulty of reaction between occluded primary amines and bulky fluorescamines arising from steric hindrance.

### 3.3. Molar Mass Measurements with GPC

The molar masses of the HECP2ks were measured by using GPC (Figure S2). Each chromatogram of HECP2k 1X, 5X, and 10X exhibited a single peak, demonstrating that synthesis of the polymers and purification by dialysis were well performed. The weight average molar mass (Mw) and PDI of OxHEC were measured as 35.63 × 10^3^ g/mol and 5.89, respectively. For HECP2k 1X, 5X, and 10X, the weight average molar mass was 13.92 × 10^3^ g/mol, 9.38 × 10^3^ g/mol, and 7.27 × 10^3^ g/mol, respectively, and their PDI values were 2.37, 2.36, and 2.25, respectively (Table S1).

The reason why the molar masses of the HECP2ks were found to be lower than that of OxHEC was thought to be due to the interaction with the column after conjugation of PEI, which can cause delay in the retention time of samples. It is well known that retention time can be affected by several factors including interaction with the column, hydrophilicity, and the three-dimensional structure of the polymer [22]. Interestingly, the Mws of HECP2ks decreased as the feed ratio of PEI2k increased. The highest Mw of HECP2k 1X was probably induced by the appropriate stoichiometric balance between two different multi-functional polymers (aldehydes of OxHEX and amines of PEI25k) in the crosslinking reaction for the synthesis of HECP2k 1X, whereas in the case of HECP2k 5X and 10X, an excess amount of PEI2k would disrupt the stoichiometric balance between them, which may have caused earlier completion of the reaction, leading to the decrease in Mws.

### 3.4. Structural Analysis with ^13^C NMR and FT-IR

Additional structural analyses for HECP2k 10X were conducted using ^13^C NMR (Figure S3) and FT-IR (Figure S4). The ^13^C NMR spectra of HECP2k 10X exhibited broad carbon peaks of the cellulose backbone at δ 68–103. The peaks of the ethylene oxide side chains were observed at δ 60–62. Additionally, the characteristic carbon peaks of PEI were observed at δ 37–58. The ^13^C NMR result also confirmed a successful synthesis of HECP2k.

The synthesis of HECP2k was also identified by FT-IR analysis. C=O peak (1740–1720 cm^−1^) was observed in the OxHEC spectrum contrary to the HEC spectrum, suggesting aldehyde formation by periodate oxidation. Then, N-H and C-N peaks were observed in both PEI2k and HECP2k spectra, meaning conjugation of PEI2k to HEC. Notably, the C=O peak disappeared in the HECP2k spectrum. These results also confirmed a successful synthesis of HECP2k.

### 3.5. pDNA Condensing Ability with Agarose Gel Electrophoresis

**Figure 2:**
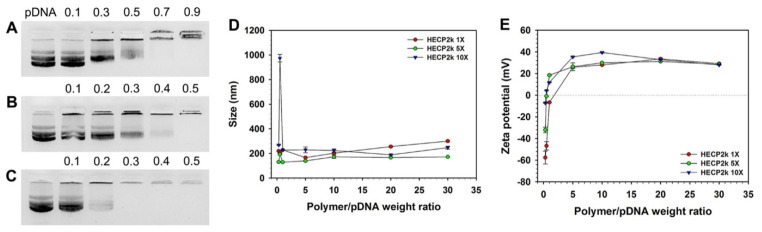
Agarose gel electrophoresis of (**A**) HECP2k 1X, (**B**) HECP2k 5X, and (**C**) HECP2k 10X. (**D**) Z-average size and (**E**) zeta-potential values of HECP2k/pDNA polyplexes.

Agarose gel electrophoresis was conducted in order to examine the pDNA condensing ability of HECP2k (Figure 2). HECP2k 10X could retard the migration of pDNA completely at a weight ratio of 0.3, but HECP2k 1X and 5X could condense pDNA at higher weight ratios of 0.9 and 0.5, respectively. This result demonstrates that HECP2k can form polyplexes with negatively charged pDNA by electrostatic interaction and the pDNA condensing ability of HECP2k increases as the conjugation ratio of PEI to HEC increases, probably due to the increase in charge density.

### 3.6. Average Size and Zeta-Potential Measurements

The transfection efficiency of gene delivery carriers can be affected by their particle sizes and surface charges. Therefore, the average particle size and zeta-potential value of HECP2k/pDNA polyplexes were measured at various weight ratios by using a Zeta-sizer. Z-average sizes of all HECP2k/pDNA polyplexes were found to be above 100 nm and below 300 nm at higher weight ratios than 1 (Figure 2D). Interestingly, a drastic increase in HECP2k 10X polyplex size was observed at a weight ratio of 0.5. This was thought to be due to aggregation by hydrophobic interaction between electrically neutralized polyplex particles at the weight ratio.

In the case of zeta-potential measurement (Figure 2E), all HECP2k polyplexes demonstrated negative zeta-potential values at a weight ratio of 0.3, and the values abruptly turned into positive values with the increase in weight ratios. They were higher than 20 mV above a weight ratio of 5. The zeta-potential values of the HECP2k 5X and 10X polyplexes became positive at lower weight ratios than the HECP2k 1X polyplexes due to higher charge densities because of higher conjugation degrees of PEI2k. These results suggest that HECP2k can form positively charged and nano-sized polyplexes with pDNA, which are needed for adsorption to a negatively charged cell membrane and efficient cellular uptake of the polyplexes [23,24].

### 3.7. Cell Metabolic Activity Analysis with MTT Assay

**Figure 3:**
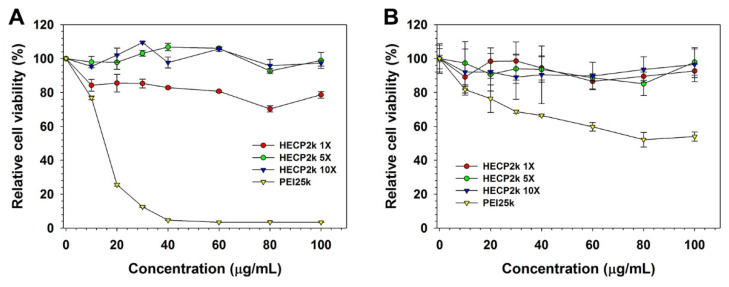
MTT assay results in (**A**) HeLa and (**B**) HepG2 cells.

The cytotoxicity of HECP2k was estimated via MTT assay (Figure 3) in HeLa and HepG2 cells. In the case of HeLa cells (Figure 3A), PEI25k demonstrated significant cytotoxicity even at low concentrations even though HECP2k 5X and 10X demonstrated negligible cytotoxicity with high RCV (>90%) at all concentrations. Interestingly, HECP2k 1X-treated cells exhibited a decrease in RCV (~80%), and this was thought to be due to the high molar mass of the polymer [25].

In HepG2 cells (Figure 3B), all the HECP2k demonstrated similarly low cytotoxicity (> 85% RCV). However, the RCV of PEI25k-treated cells decreased gradually, contrary to the HeLa cell result. This may be due to the resistance and low susceptibility of HepG2 cells to toxic agents [26,27].

### 3.8. Transfection Efficiency Measurement with Luciferase Transgene Expression Assay

**Figure 4:**
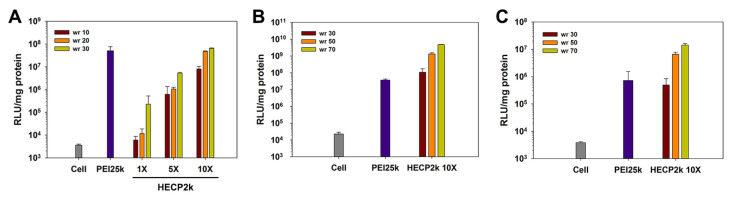
(**A**) Luciferase assay results of HECP2k polyplexes in HeLa cells at the various weight ratios (weight ratio, wr = 10, 20, or 30). Luciferase assay results of HECP2k 10X polyplexes in HeLa cells at the various weight ratios in (**B**) serum-free conditions and (**C**) serum conditions. PEI25k polyplexes (wr = 1) were used as control.

The transfection efficiency of HECP2k was examined via luciferase transgene expression assay. pCN-Luci (pDNA) was used as a reporter gene that expressed luciferase protein. First, in order to compare the transfection efficiency of HECP2k 1X, 5X, and 10X (polyplex weight ratios = 10~30), the assay was performed in serum-free conditions in HeLa cells. The transfection efficiency of all HECP2k polymers was increased with the increase in polyplex weight ratios and PEI conjugation ratios (Figure 4A). Although HECP2k 1X and 5X demonstrated lower transfection efficiency than PEI25k, HECP2k 10X demonstrated high transfection efficiency similar to PEI25k at a weight ratio of 30. Therefore, later experiments were conducted with HECP2k 10X.

After confirmation of HECP2k 10X as an optimized carrier, additional transfection experiments were performed at the extended weight ratios of 30, 50, and 70 (where HECP2k 10X exhibited minimum cytotoxicity) in serum-free (Figure 4B) or serum conditions (Figure 4C) in HeLa cells. The transfection efficiency of HECP2k 10X was 35–127 times higher than that of PEI25k at weight ratios of 50 and 70 in serum-free conditions. Even in serum conditions (weight ratio of 50 and 70), the transfection efficiency of HECP2k 10X was 9–19 times higher than that of PEI25k. These results indicate that HECP2k 10X has superior transfection efficiency.

### 3.9. Cellular Uptake Measurements with Flow Cytometry

**Figure 5:**
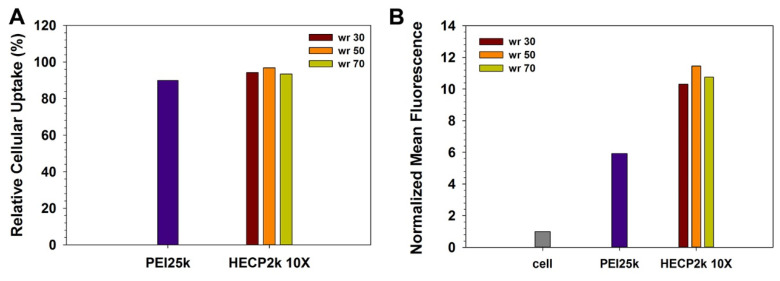
Flow cytometry results of HECP2k 10X polyplexes in HeLa cells at the various weight ratios. (**A**) Relative cellular uptake and (**B**) normalized mean fluorescence unit of polyplexes. PEI25k polyplexes (wr = 1) were used as control.

Cellular uptake efficiency was estimated by using polyplexes formed with YOYO-1 iodide-labeled pDNA in HeLa cells (Figure 5). In the case of relative cellular uptake (%), HECP2k 10X and PEI25k demonstrated similar populations of fluorescent cells (about 90%). However, the normalized mean fluorescence of HECP2k 10X polyplexes-treated cells was 1.7–1.9 times higher than PEI25k polyplexes-treated cells. One possible explanation for this high cellular uptake efficiency of HECP2k 10X polyplexes is the water retainability of HEC. Water retention and hydration of HEC can cause osmotic pressure near the cellular membrane [28]. It has been reported that osmotic stress to cells can induce caveolae-mediated endocytosis via the stimulation of caveolin-1 (Cav-1) [29,30]. This also would lead to high transfection efficiency of HECP2k 10X.

### 3.10. Intracellular Trafficking Observation with CLSM

**Figure 6:**
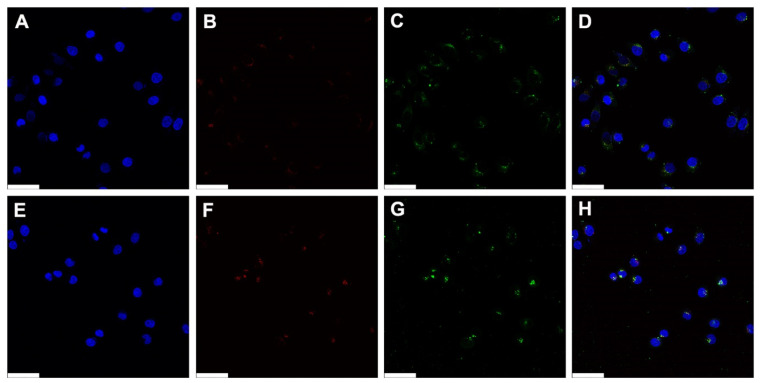
CLSM images of PEI25k polyplexes (**A**–**D**) and HECP2k 10X polyplexes (**E**–**H**) in HeLa cells. DAPI (blue) stained nuclei (**A**,**E**) and Lysotracker red DND-99 (red) stained acidic organelles (**B**,**F**). pDNA was labeled using YOYO-1 (green, (**C**,**G**)). (**D**,**H**) show merged images (scale bar = 50 μm). After 4 h of treatments, cells were visualized after 4 h of further incubation.

The intracellular trafficking of polyplexes was observed via CLSM (Figure 6). pDNA was labeled with YOYO-1 iodide (green), acidic organelles were labeled with Lysotracker red DND-99 (red), and nuclei were labeled with DAPI (blue). Considering cytotoxicity, transfection efficiency, and cellular uptake efficiency, the weight ratio of 50 was regarded as an optimized weight ratio for HECP2k 10X polyplexes.

Intracellular uptake of the polyplexes was observed in most cells in both PEI25k and HECP2k 10X polyplexes. This result corresponds to the previous flow cytometry result that polyplexes-treated cells demonstrated an about 90% fluorescent population. In the case of PEI25k, the distribution of green fluorescence from polyplexes was mainly located in cytosol at 0 h, 2 h, and 4 h after treatments (Figures S5A, S5C, Figure 6 and Figure S6A). Meanwhile, HECP2k 10X polyplexes exhibited green fluorescence in the cytosol right after treatment (Figure S5B). However, an accumulation of green fluorescent polyplexes was found near and inside of nuclei at 2 h (Figure S5D) and 4 h (Figure 6H and Figure S6B) after treatments. In addition, there were less cells containing the co-localized signals between green and red fluorescence in comparison with PEI25k polyplexes-treated cells, which means that HECP2k 10X polyplexes escaped the endosome rapidly (Figure S6B). This may be probably due to the high endosome buffering capacity and osmotic stress of HECP2k 10X. These results demonstrate that HECP2k 10X exhibits superior and more efficient intracellular trafficking behaviors than PEI25k.

### 3.11. Serum Stability Test

It has been reported that serum proteins interact with cationic polyplexes, leading to the elimination of polyplexes and a following reduction in transfection efficiency [31]. Therefore, it is important to decrease serum interaction and improve the serum stability of polyplexes.

**Figure 7:**
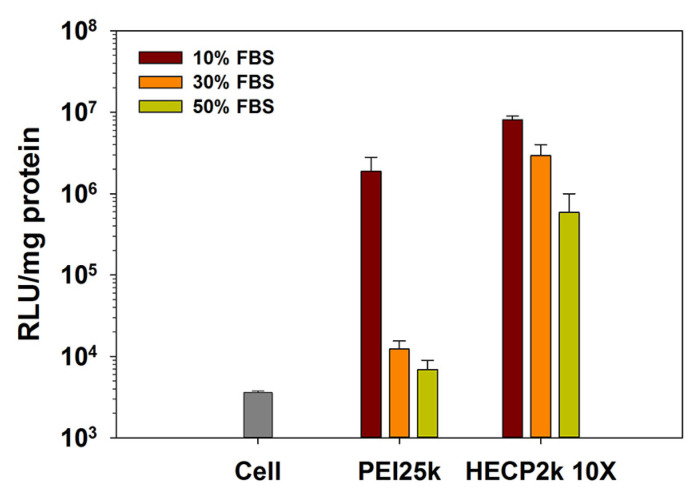
Serum stability test results of HECP2k 10X polyplexes (wr = 50) via luciferase assay. PEI25k polyplexes (wr = 1) were used as control.

To estimate serum stability, transfection efficiencies of HECP2k 10X polyplexes were measured in 10%, 30%, and 50% FBS conditions. As shown in Figure 7, the transfection efficiency of PEI25k decreased drastically as the concentration of FBS increased, and it decreased to a level similar to the untreated cell group value in 50% FBS conditions. However, in the case of HECP2k 10X polyplexes, the tendency of the transfection efficiency decrease was alleviated compared to PEI25k polyplexes. It is noteworthy that HECP2k retained a transfection efficiency up to 85.4 times higher than PEI25k polyplexes even at 50% FBS concentration. This result demonstrates that HECP2k 10X has great serum stability. It is thought that ethylene oxide groups of HEC hamper the interaction with serum protein by steric blocking.

### 3.12. Drug Loading Contents and Drug Loading Efficiency of HECP2k 10X

The anti-cancer agent Doxorubicin (Dox) was encapsulated in HECP2k 10X using the dialysis method [32]. After lyophilization, Dox-loaded HECP2k 10X@Dox powder was obtained. Despite the hydrophobicity of Dox, it could be loaded by interaction with the polymer through hydrogen bonding or physical embedding [32,33,34]. The drug loading contents and efficiency of HECP2k 10X were obtained by measurement of Dox absorbance at 570 nm (water:DMSO = 9:1, *v*/*v*). When the mixing ratio (Dox/HECP2k 10X, *w*/*w*) was 3/10, the drug loading efficiency (DLE) and drug loading content (DLC) were 41.49% and 13.83%, respectively.

### 3.13. Average Size and Zeta-Potential Measurements

The average size and zeta-potential value of HECP2k 10X/siRNA polyplexes and HECP2k 10X@Dox/siRNA complexes at an optimized weight ratio of 50 were measured using a Zeta-sizer. The average size and zeta-potential value of HECP2k 10X/siRNA were 153.7 ± 3.0 nm and 14.7 ± 2.4 mV, respectively. After doxorubicin encapsulation, the average size and zeta-potential value were increased to 241.3 ± 0.1 nm and 17.0 ± 0.8 mV, respectively, which were still a proper size and surface charge for cellular uptake.

### 3.14. Anti-Cancer Effect Analysis with MTT Assay

The anti-cancer effect of the HECP2k 10X@Dox/siRNA complexes were investigated via MTT assay in HeLa and HepG2 cells in order to identify the combinatorial effect of co-delivery of Dox and Bcl-2 siRNA (Figure S6). Dox exhibits anti-cancer effects by disrupting DNA repair and generating free radicals, while Bcl-2 mRNA can be cleaved by the RNA-induced silencing complex (RISC) composed of Bcl-2 siRNA. Bcl-2 proteins are especially overexpressed in tumor cells, inhibiting cell death and contributing to the abnormal growth of the tumor [35,36]. Therefore, downregulation of Bcl-2 protein can result in apoptosis induction.

**Figure 8:**
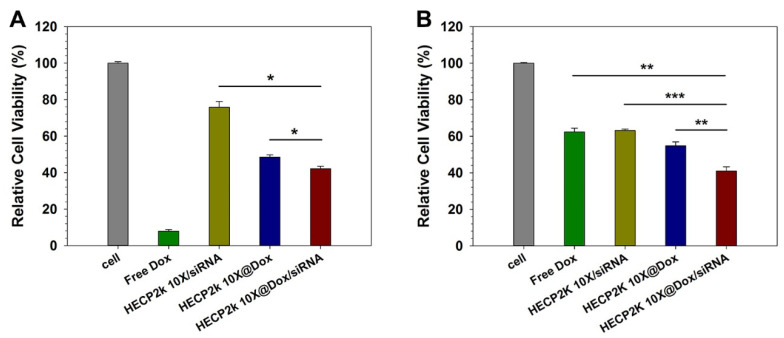
Relative cell viability for free Dox, HECP2k 10X/siRNA, HECP2k 10X@Dox/siRNA complexes at a high concentration of Dox and siRNA in (**A**) HeLa and (**B**) HepG2 cells. Statistical analysis was performed through one-way ANOVA and Bonferroni post hoc test. Statistical significance is presented as follows (*p* < 0.05 *, *p* < 0.01 **, *p* < 0.001 ***).

Each concentration of loaded Dox and siRNA of HECP2k 10X@Dox/siRNA complexes is shown in Table S2. In HeLa cells (Figure S7A), free Dox demonstrated a strong cancer cell-killing effect. Free siRNA could not induce any cytotoxic effect in HeLa cells because of very low membrane permeation efficiency caused by the absence of the carrier. The PEI25k/siRNA polyplexes-treated group also exhibited a low cancer cell-killing effect. However, in the case of HECP2k 10X/siRNA polyplexes, these exhibited a higher cancer cell-killing effect compared to PEI25k/siRNA polyplexes, probably due to the higher transfection efficiency of HECP2k 10X compared to PEI25k. HECP2k 10X@Dox-treated cells exhibited 48.5% cell viability and HECP2k 10X@Dox/siRNA complexes could reduce the cell viability to 42.1% at a high concentration (15 μg/mL) (Figure 8A).

Contrary to HeLa cells, free Dox-treated HepG2 cells demonstrated above 60% cell viability even at a high concentration (15 μg/mL) (Figure S7B and Figure 8B). Several studies have reported that HepG2 cells are less sensitive to Dox than HeLa cells [37,38]. Except for free siRNA, the viability of other samples-treated cells decreased as the concentration increased, showing their cancer cell-killing effect. Among them, HECP2k 10X@Dox/siRNA complexes were the only group that exhibited a cell viability of less than 50%. As reported in recent studies that nanoparticles with chemotherapeutics can evade drug efflux pumps [39], increasing intracellular drug concentration, it was thought that HECP2k 10X@Dox/siRNA nano-complexes could overcome the lower sensitivity of HepG2 cells, leading to a high anti-cancer effect.

Median effect analysis was also conducted to construct the median effect plot [40,41] for the identification of drug synergism. Briefly, dose–effect curves were constructed based on previous MTT assay results (Figure S8A,B). The dose and effect relations could be linearized by plotting log (dose) vs. log [fraction affected (f_a_)/fraction unaffected (f_u_)] (Figure S8C–F). Based on the median effect equation, the theoretical minimum of constructing a dose–effect curve requires only two data points. The median effect equation is shown below.

$$\frac{fa}{fu}={\left(\frac{D}{Dm}\right)}^{m}$$

fa= fraction of system affected;

$fu=\left(1-fa\right)=$ fraction unaffected;

D= dose required to produce fa;

Dm= dose required to produce median effect, i.e., IC50;

m= dynamic order (shape).

Based on the median effect plots (Figure S8C–F), the combination index (CI) was calculated (Figure S9). Values of CI > 1, = 1, < 1 indicate antagonism, additive effect, and synergism, respectively. As for HeLa cells (Figure S9A), synergism was found at 0.2 or less affected fractions, but there was enormous antagonism above that. This result was thought to be due to the non-drug resistance of HeLa cells and the strong cytotoxicity of Dox itself. Since the affected fraction below 0.2 is not valuable for cancer treatment, it is difficult to expect a synergistic effect of HECP2k 10X@Dox/siRNA complexes for HeLa cells. However, for HepG2 cells (Figure S9B), synergism was shown at 0.2 or higher affected fractions. These results demonstrate that co-delivery of Dox with Bcl-2 siRNA can exhibit synergism that may be due to the diminishing of MDR characteristics by down-regulation of Bcl-2 proteins [42,43,44]. Therefore, HECP2k 10X for co-delivery of Dox with Bcl-2 siRNA may be considered a therapeutic delivery system with great potential.

### 3.15. Apoptosis Induction Test with Annexin V Staining

**Figure 9:**
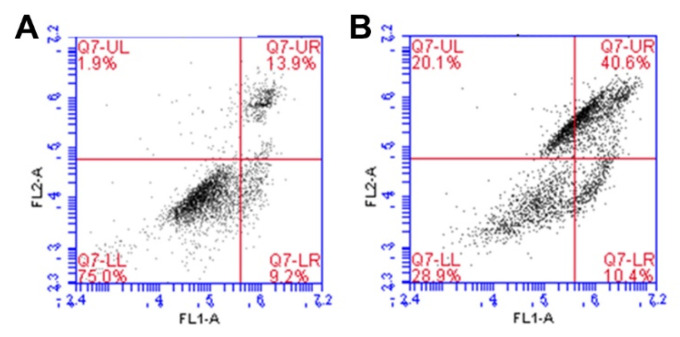
Flow cytometry results via annexin V staining of (**A**) untreated cells and (**B**) HECP2k 10X@Dox/siRNA complexes-treated cells. (FL1: Alexa Fluor 488, FL2: PI).

In order to verify that HECP2k 10X@Dox/siRNA complexes induce cell death through apoptosis, Annexin V staining was conducted (Figure 9). The human vascular anticoagulant annexin V is a Ca^2+^-dependent phospholipid-binding protein with high affinity for the anionic phospholipid phosphatidylserine (PS). In normal healthy cells, PS is located on the cytoplasmic surface of the plasma membrane. During apoptosis, structural collapse of plasma membrane occurs and this leads PS to translocate from the inner to the outer side of the plasma membrane. Therefore, PS on the outer surface of the cell has been used as a pro-apoptosis marker [45].

To stain the pro-apoptotic cells, Alexa fluor 488 was used (FL1). Dead cells were stained using propidium iodide (PI, FL2). As shown in Figure 9, in the untreated control group (Figure 9A), 75.0% of the cells were live, 9.2% were undergoing apoptosis, and 15.8% were spontaneously dead, compared to the HECP2k 10X@Dox/siRNA complexes-treated group (Figure 9B), where only 28.9% of the cells were live and 60.7% were dead. Notably, 10.4% of the cells were found to be undergoing apoptosis, or other cell populations exhibited completed apoptosis. This result demonstrates that HECP2k 10X@Dox/siRNA complexes could kill cancer cells by inducing apoptosis as expected.

## 4. Conclusions

In this work, HEC was utilized as a template polymer for drug/gene co-delivery systems due to several advantages such as its high biocompatibility and water solubility. PEI2k was successfully conjugated to HEC in a controllable manner via simple periodate oxidation and reductive amination. The synthesized HECP2ks condensed pDNA into positively charged and nano-sized particles. HECP2ks exhibited low cytotoxicity and HECP2k 10X exhibited a higher transfection efficiency than PEI25k even in serum conditions, showing its high serum stability because of ethylene oxide side chains. Additionally, the endosome buffering ability of PEI2k and osmotic stress caused by HEC of HECP2k 10X facilitated cellular uptake and endosome escape, leading to efficient transfection. HECP2k 10X also loaded Dox and Bcl-2 siRNA, forming nano-particles (HECP2k@Dox/siRNA). Finally, it was found that HECP2k 10X@Dox/siRNA complexes caused synergistically enhanced anti-cancer effects of Dox and Bcl-2 siRNA in cancer cells via induction of apoptosis. Consequently, it is concluded that HECP2k possesses great potential as a promising Dox/Bcl-2 siRNA co-delivery carrier.

## Data Availability

Data available on request due to restrictions eg privacy or ethical.

## Supplementary Materials

The following supporting information can be downloaded at: https://www.mdpi.com/article/10.3390/pharmaceutics15020708/s1, Figure S1: Synthesis scheme of HECP2k; Figure S2: GPC chromatograms of polymers; Figure S3: ^13^C NMR spectra of HECP2k 10X; Figure S4: FT-IR spectra of polymers; Figure S5: CLSM images of PEI25k/pDNA and HECP2k 10X/pDNA polyplexes in HeLa cells; Figure S6: Magnified CLSM images of PEI25k/pDNA and HECP2k 10X/pDNA polyplexes in HeLa cells; Figure S7: MTT assay results showing anti-cancer effects of free dox, free siRNA, PEI25k/siRNA, HECP2k 10X@Dox, HECP2k 10X/siRNA, and HECP2k 10X@Dox/siRNA complexes in HeLa and HepG2 cells; Figure S8: Dose–effect curves of Dox and siRNA in HeLa and HepG2 cells. Median effect plots for Dox in HeLa and HepG2 cells. Median effect plots for Bcl-2 siRNA in HeLa and HepG2 cells; Figure S9: Combination index (CI) of HECP2k 10X@Dox/siRNA complexes based on the median effect plots for HeLa and HepG2 cells; Table S1: Molecular weight analysis result of polymers via GPC; Table S2: Dox and Bcl-2 siRNA concentration for each condition.
