# Supplementary material for: Polyethylenimine-Conjugated Hydroxyethyl Cellulose for Doxorubicin/Bcl-2 siRNA Co-Delivery Systems
Source: Pharmaceutics. 2023 Feb 20;15(2):708. doi: 10.3390/pharmaceutics15020708 (PMC9965717; doi:10.3390/pharmaceutics15020708)
Supplement: Supplementary file 1 [file pharmaceutics-15-00708-s001.zip › pharmaceutics-2160676-supplementary.pdf]

Supplementary Materials

Figure S1. Synthesis scheme of HECP2k

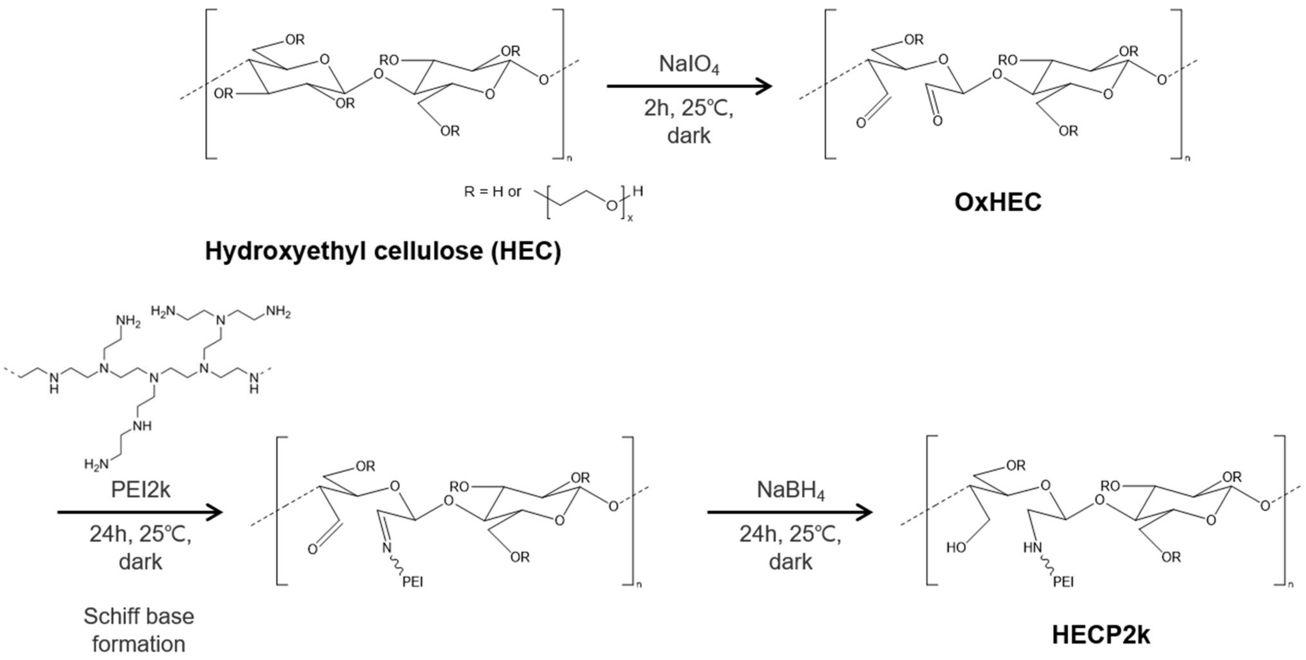

Figure S2. GPC chromatograms of polymers. Each sample was prepared at a concentration of 10 mg/mL. 1% formic acid was used as an eluent. The assay was run at 0.6 mL/min of flow rate.

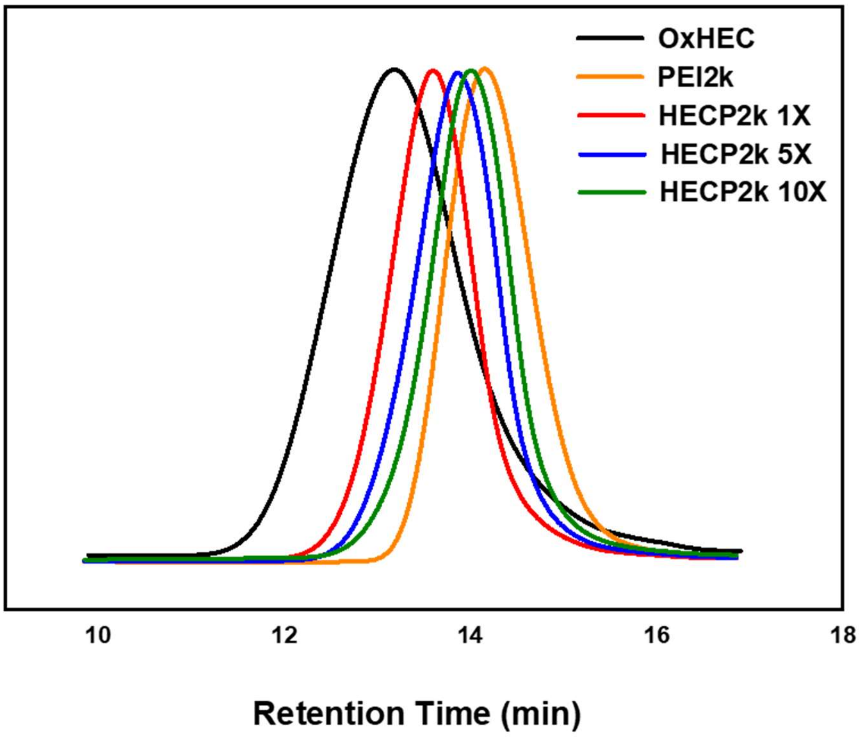

Figure S3. ^{13}C NMR spectra of HECP2k 10X.

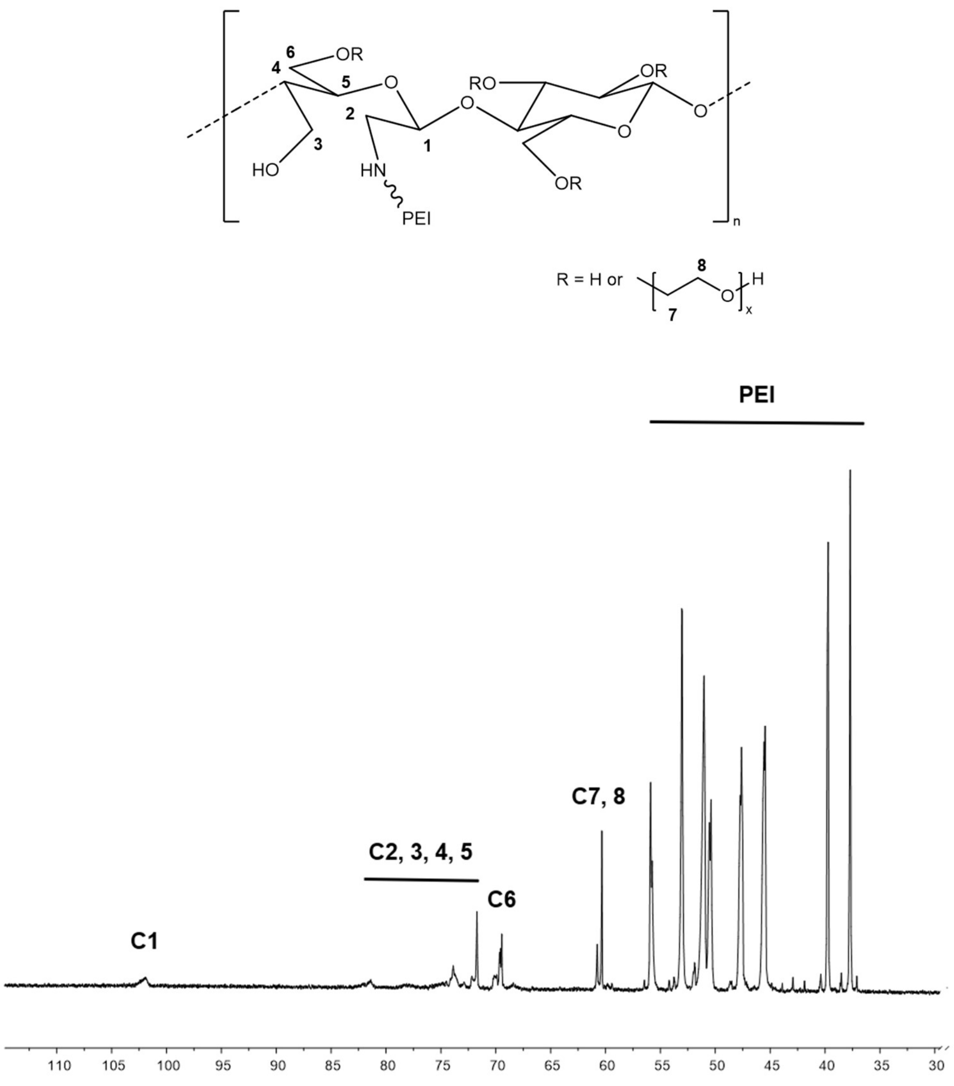

Figure S4. FT-IR spectra of polymers.

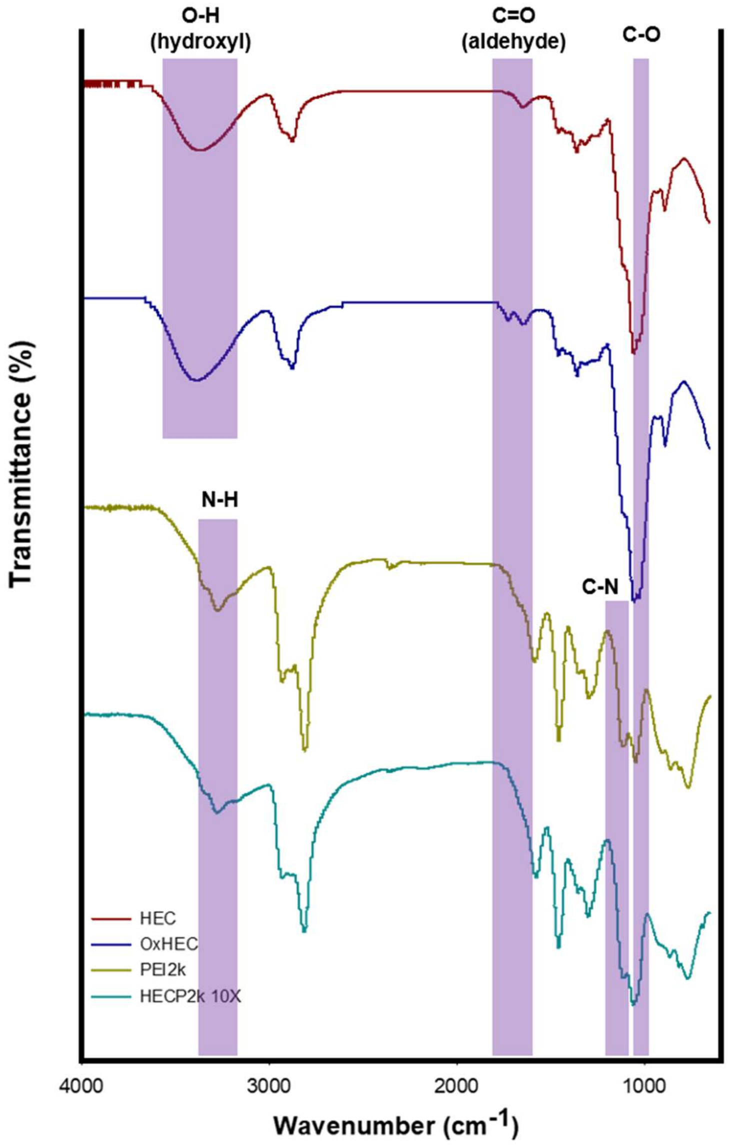

Figure S5. CLSM images of PEI25k/pDNA (A, C) and HECP2k 10X/pDNA (B, D) polyplexes in HeLa cells. pDNA was labeled by YOYO-1 (green). Nuclei and acidic organelles were stained by DAPI (blue) and Lysotracker red DND-99 (red), respectively. After 4 h of treatment, cells were visualized after further incubation of (A, B) 0 h and (C, D) 2 h.

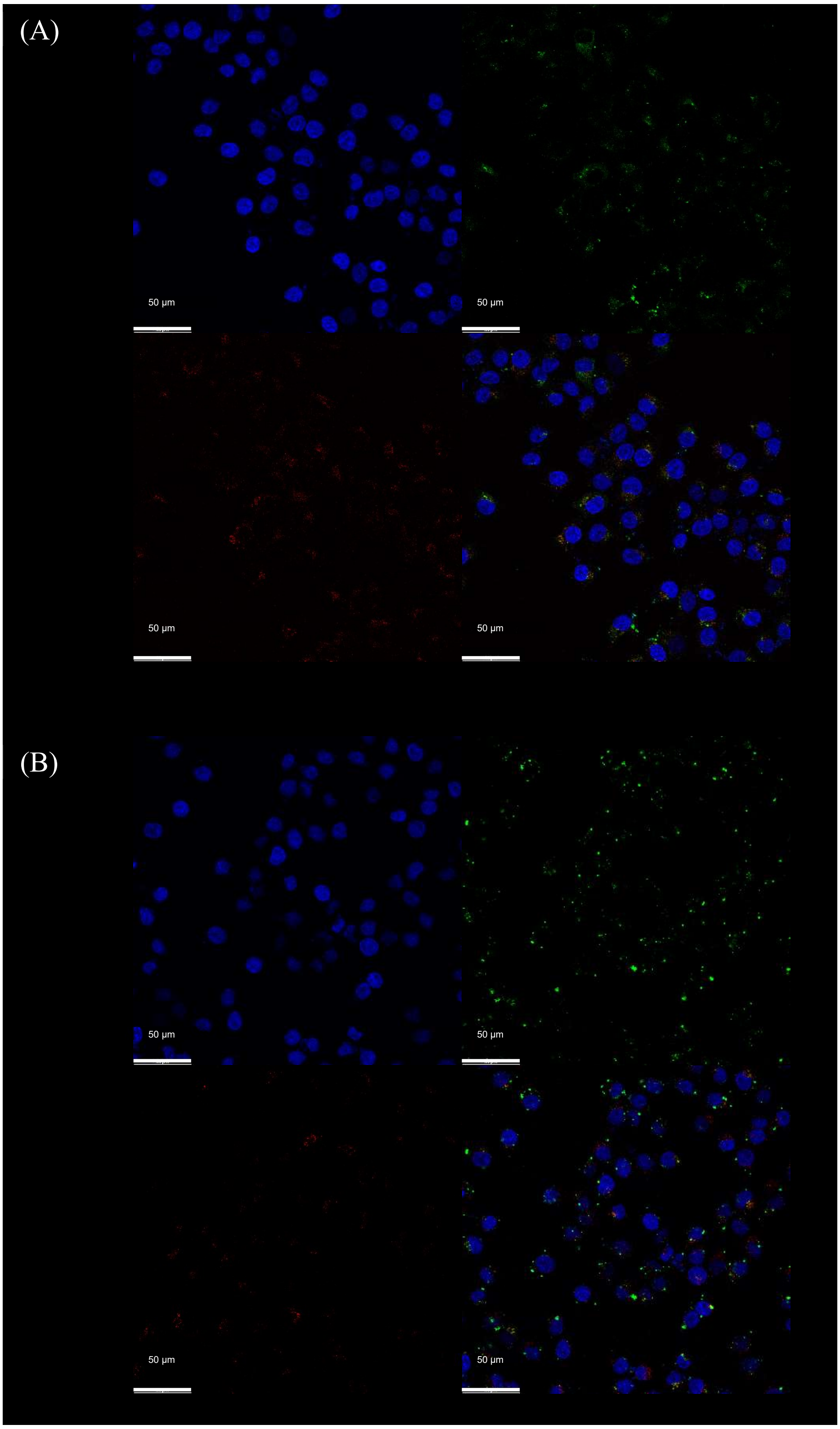

Figure S5. Continued.

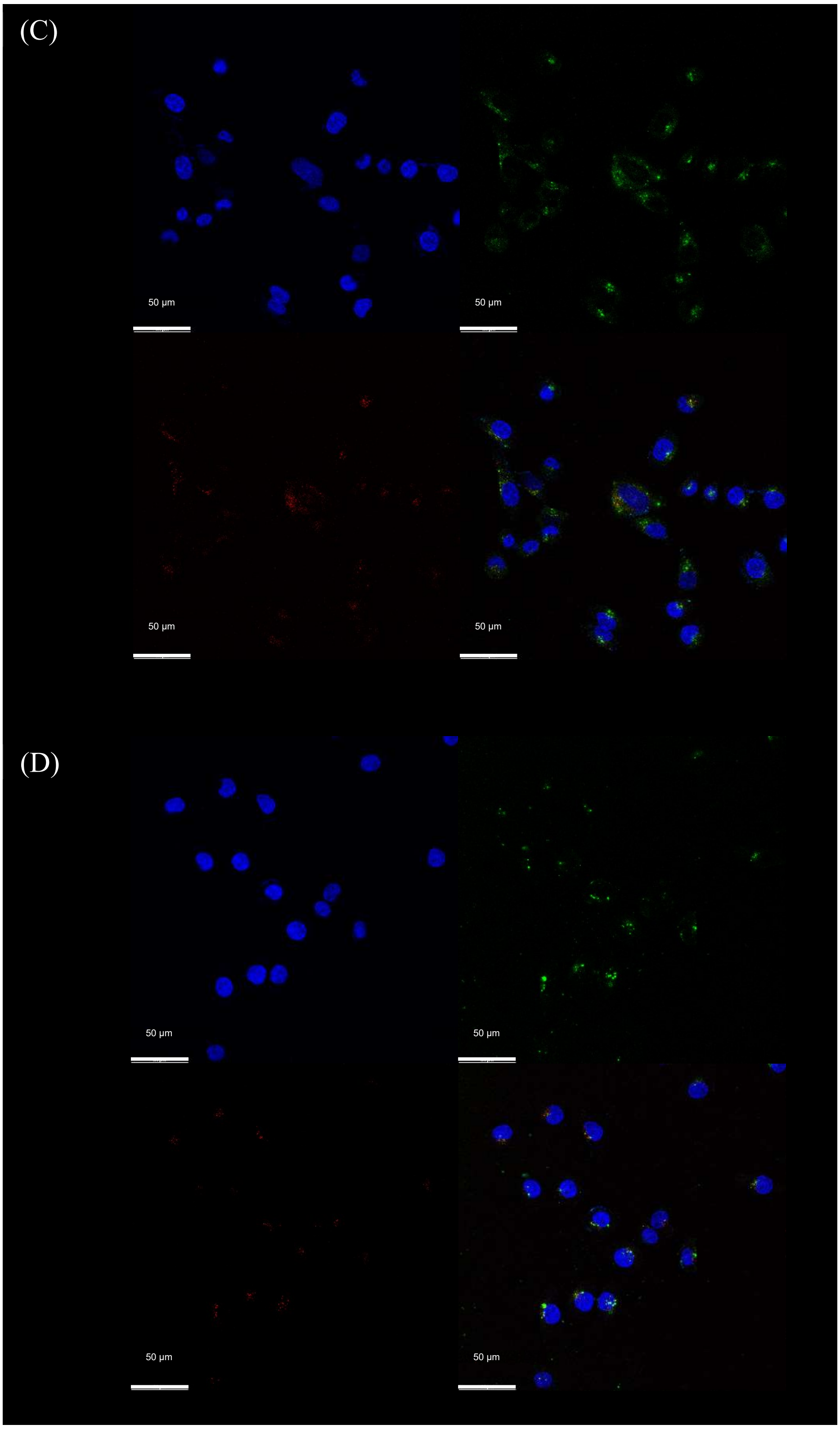

Figure S6. Magnified CLSM images of PEI25k/pDNA (A) and HECP2k 10X/pDNA (B) polyplexes in HeLa cells. After 4 h of treatment, cells were visualized after further incubation of 4 h (scale bar = 50 μm).

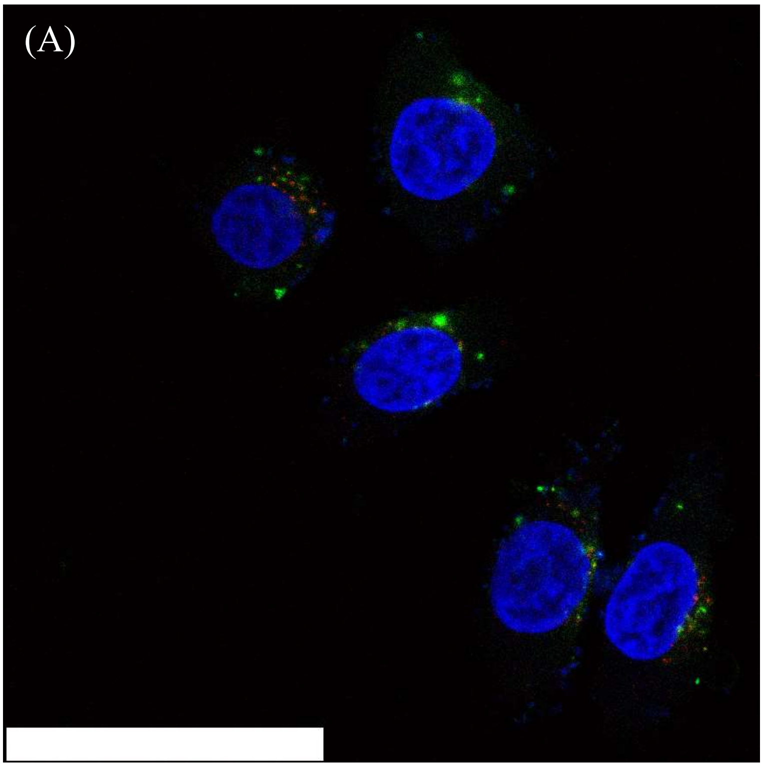

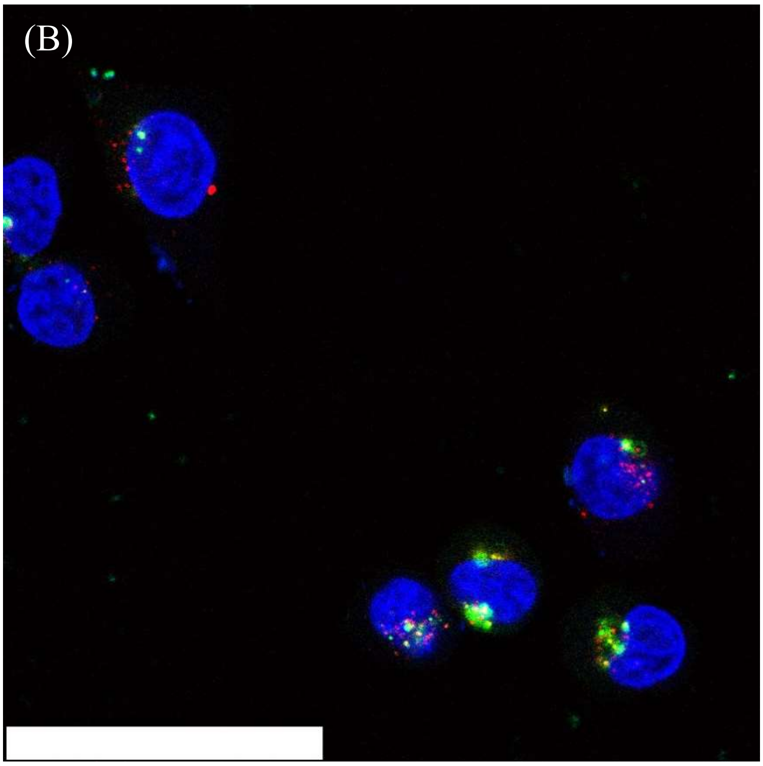

Figure S7. MTT assay results showing anti-cancer effects of free dox, free siRNA, PEI25k/siRNA, HECP2k 10X@Dox, HECP2k 10X/siRNA, and HECP2k 10X@Dox/siRNA complexes in (A) HeLa and (B) HepG2 cells.

(A)

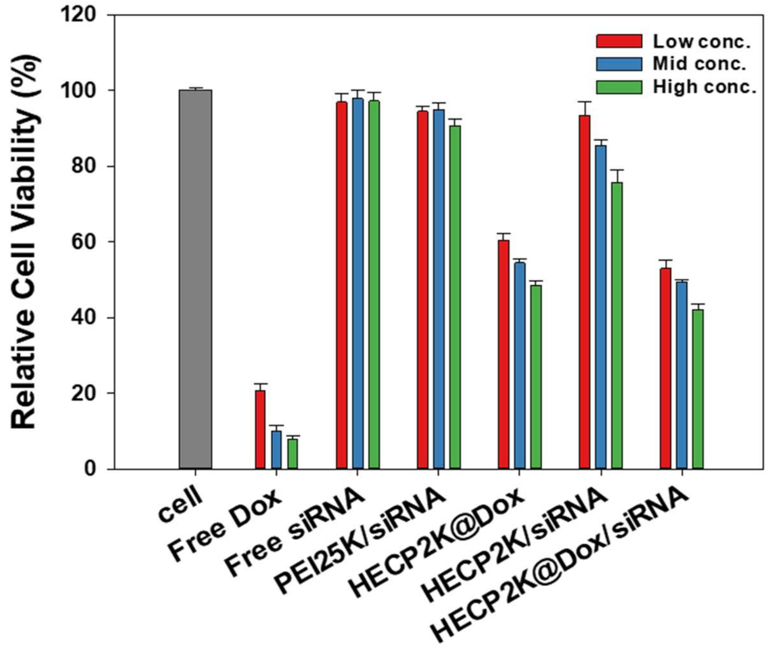

(B)

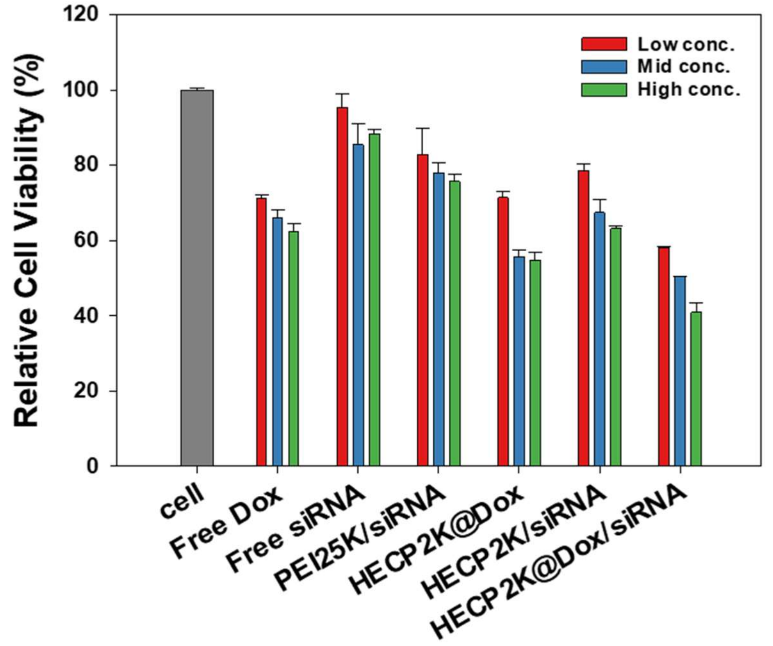

Figure S8. Dose-effect curves of Dox and siRNA in (A) HeLa and (B) HepG2 cells. Median effect plots for Dox in (C) HeLa and (D) HepG2 cells. Median effect plots for Bcl-2 siRNA in (E) HeLa and (F) HepG2 cells.

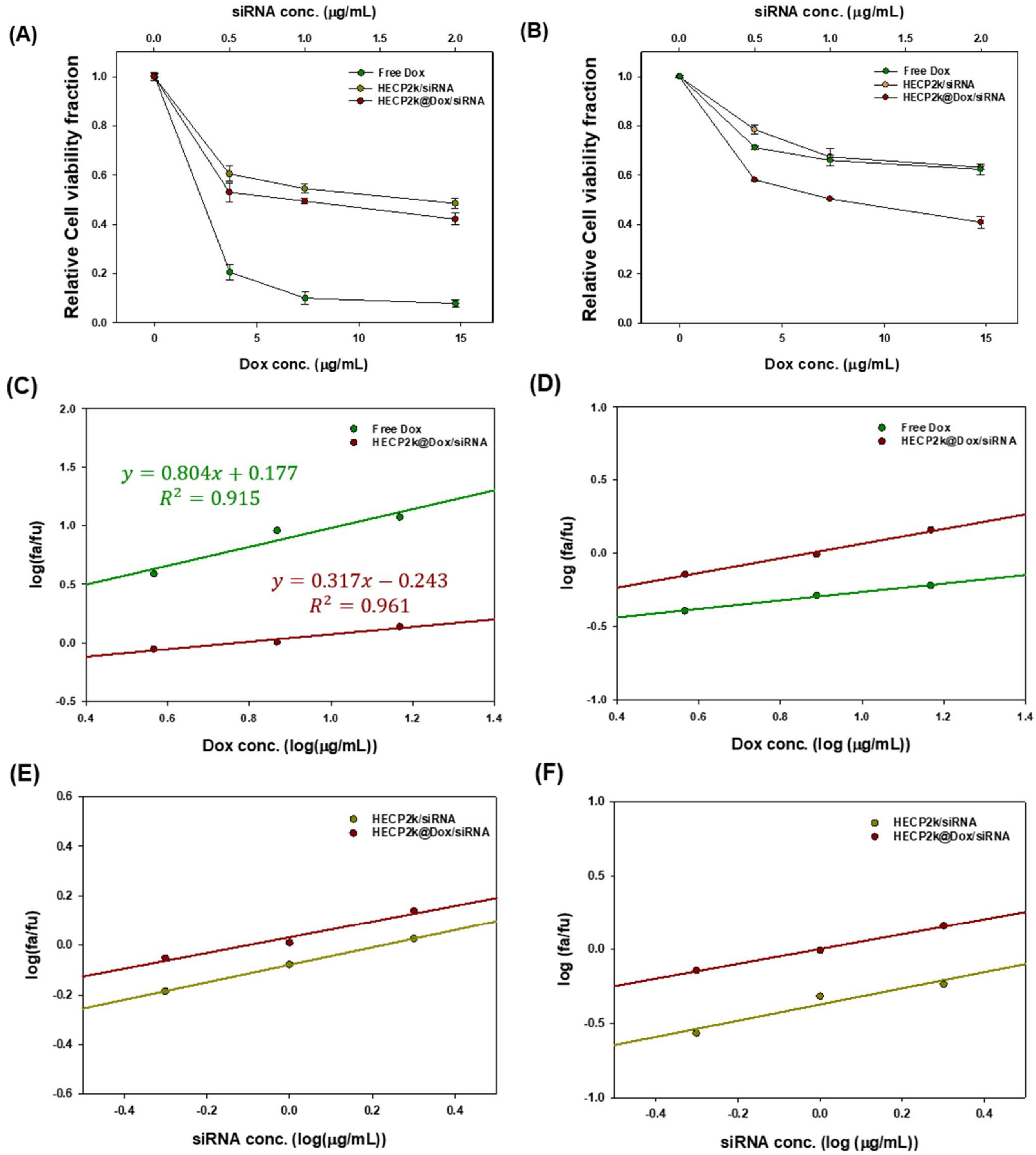

Figure S9. Combination index (CI) of HECP2k 10X@Dox/siRNA complexes based on the median effect plots for (A) HeLa and (B) HepG2 cells.

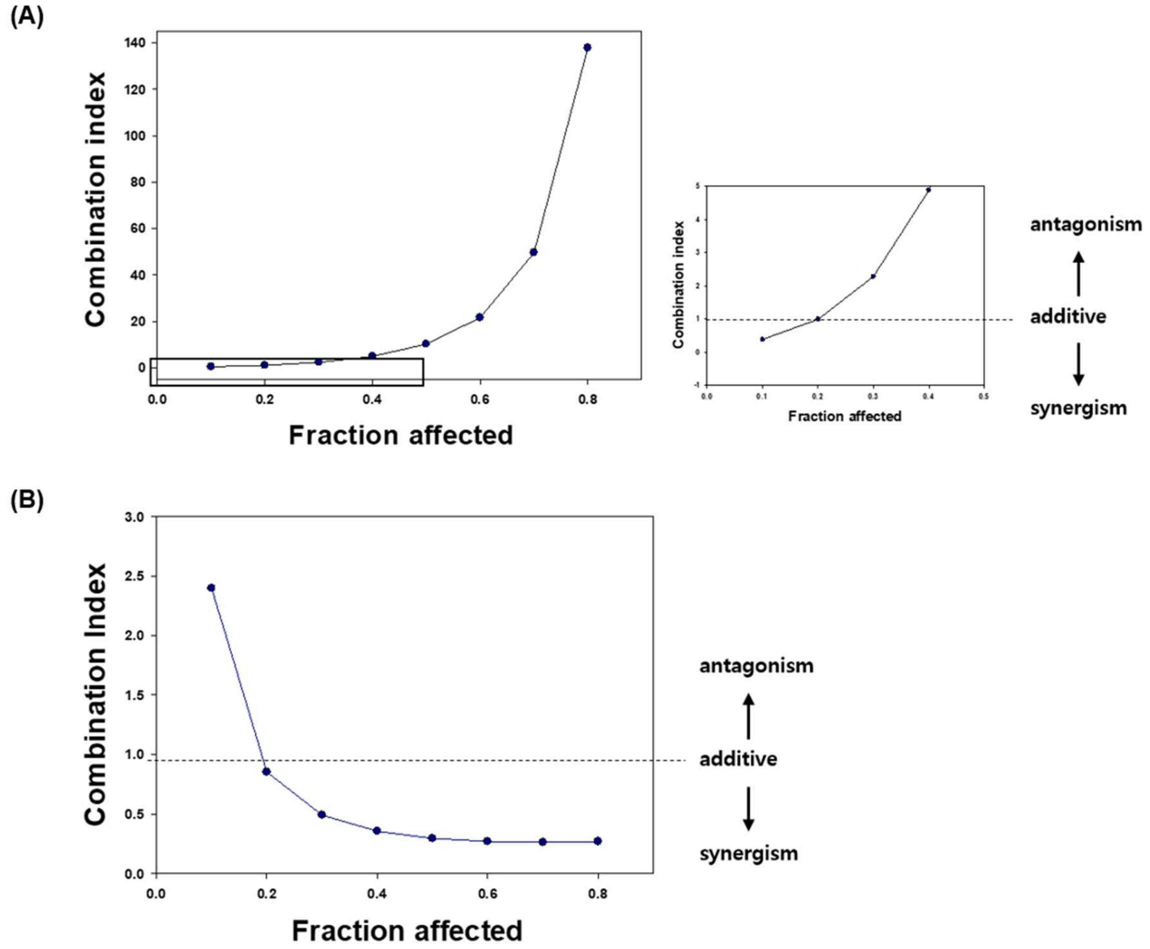

Table S1. Molecular mass analysis result of polymers by GPC.

Sample	Mn (GPC, kg/mol)	Mw (GPC, kg/mol)	PDI	RT (min)
OxHEC	6.05	35.63	5.89	13.21
PEI2k	1.95	4.61	2.36	14.25
HECP2k 1X	5.88	13.92	2.37	13.70
HECP2k 5X	3.97	9.38	2.36	13.95
HECP2k 10X	3.23	7.27	2.25	14.09

Table S2. Dox and Bcl-2 siRNA concentration for each condition.

HECP2k@Dox	Dox (μg/mL)	siRNA (μg/mL)
Low conc.	3.7	0.5
Mid conc.	7.4	1
High conc.	14.8	2
